# Conservation genetics of *Notelaea lloydii* (Oleaceae) in south‐eastern Queensland, Australia

**DOI:** 10.1002/ece3.10895

**Published:** 2024-02-07

**Authors:** Chapa G. Manawaduge, James Ryan, Matthew J. Phillips, Susan Fuller

**Affiliations:** ^1^ School of Biology and Environmental Sciences Queensland University of Technology Brisbane Queensland Australia; ^2^ Present address: CSIRO Health and Biosecurity Acton ACT Australia

**Keywords:** DArTseq, inbreeding, native olives, population structure, reproductive success, threatened species

## Abstract

Habitat fragmentation can increase the chance of population bottlenecks and inbreeding, and may ultimately lead to reduced fitness and local extinction. *Notelaea lloydii* is a native olive species endemic to Australia and listed as vulnerable due to its restricted distribution. A recent molecular systematics study has revealed there might be some geographic structuring among *N. lloydii* populations. Therefore, we undertook a genome‐wide single nucleotide polymorphism (SNP) analysis to determine levels and patterns of genetic diversity, inbreeding and gene flow within and among *N. lloydii* populations in south‐eastern Queensland. Furthermore, as the reproductive phase of a plant's life history has a profound influence on genetic diversity, life history reproductive traits were also studied. Our SNP analysis revealed low genetic diversity, inbreeding and significant genetic structuring even among proximate populations. Results of a flower and fruit bagging experiment in two consecutive seasons revealed that *N. lloydii* produced many flowers but only a few fruits survived to maturity. There were no differences in bagged and un‐bagged flowering and fruiting rates, and therefore, we conclude that the high fruit abortion rate was probably due to inbreeding depression and/or suboptimal conditions, rather than pollinator availability and insect attack. Overall, results of this study indicate that the populations of *N. lloydii* are small, inbred and genetically isolated and represent unique management units that require local conservation management due to ongoing threats associated with urbanisation.

## Graphical abstract

We have conducted a genome‐wide single nucleotide polymorphism (SNP) analysis to determine levels and patterns of genetic diversity, inbreeding and gene flow within and among the populations of one of the threatened native species, *Notelaea lloydii*, restricted in south‐eastern Queensland. Our SNP analysis revealed low genetic diversity, inbreeding and significant genetic structuring even among proximate populations, and conservation implications are discussed in the manuscript.

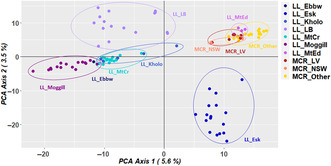

## 1 INTRODUCTION

Habitat loss and fragmentation are globally significant causes of biodiversity loss (Fahrig, 2003; Krauss et al., 2010; Wilson et al., 2016). As a result, previously widespread and connected plant populations can become small and isolated (Burrough et al., 2018; Field et al., 2008; Hobbs & Yates, 2003; Young et al., 1996). While conservation strategies for threatened plant species generally rely on protection of habitats, maintenance of genetic diversity and gene flow is critical for long‐term persistence and should be factored into species conservation plans (Bezemer et al., 2019; Ottewell et al., 2016; Ralls et al., 2018). To maintain sufficient gene flow to prevent loss of genetic variation and inbreeding, plants rely on their pollination and seed dispersal mechanisms. Fragmentation and restricted population size can increase the chance of inbreeding (Leimu et al., 2010; Lowe et al., 2015) and the effects of genetic drift, which in turn can increase homozygosity and expression of deleterious alleles in a population, leading to reduced fitness and population bottlenecks (Breed et al., 2012). Habitat fragmentation can also alter the relative abundance of cross‐compatible species within an area, which could increase the potential for inter‐specific gene flow that results in the production of hybrids (Butcher et al., 2005; Field et al., 2008; Keppel et al., 2011).

**FIGURE A1:**
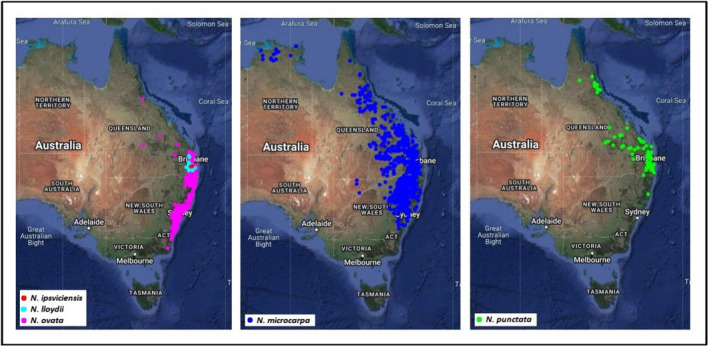
Distribution maps of the two threatened (*N. ipsviciensis* and *N. lloydii*) and three common (*N. ovata*, *N. microcarpa* and *N. punctata) Notelaea* species in Australia (*Source*: Atlas of living Australia).

*Notelaea lloydii* (Guymer) is an endemic native olive species, currently listed as a threatened species due to its small population and restricted geographic distribution in south‐eastern Queensland (SE‐QLD), Australia (Appendix 1: Figure A1), a region subject to ongoing urban development (*Approved conservation advice for Notelaea lloydii*, 2008). It was first described as a new species by Guymer (1987), and at that time, it was known only from two locations that were 60 km apart. Several other disjunct populations have since been identified within an area of approximately 3700 km^2^ in SE‐QLD (*Approved conservation advice for Notelaea lloydii*, 2008; Halford, 1998), resulting in *N. lloydii* being listed as vulnerable (Environment Protection and Biodiversity Conservation Act, 1999). A recent molecular systematics study of the genus *Notelaea* has revealed that another *Notelaea* species in the vicinity, *N. ipsviciensis* W. K. Harris, is a hybrid between *N. lloydii* and *N. ovata* (Manawaduge et al., 2022). Molecular data have revealed that hybrid backcrossing with *N. lloydii* is occurring at the site where they are sympatric. Hybridisation can reduce the viability of small populations through demographic and genetic swamping (Ellstrand & Elam, 1993; Ellstrand & Rieseberg, 2016) and may ultimately influence the time to local extinction (Wolf et al., 2001). Furthermore, this study did not find reciprocal monophyly of *N. lloydii* and *N. microcarpa*, i.e., two clades of *N. lloydii* were nested within different parts of the *N. microcarpa* clade, but some phylogeographic structure was reported (Manawaduge et al., 2022). Moreover, *N. lloydii* populations are small, each with less than 30 individuals, and limited recruitment has been observed (*Approved conservation advice for Notelaea lloydii*, 2008); therefore, further population genetic analysis is required.

In order to investigate the genetic diversity, inbreeding and spatial genetic structure among *N. lloydii* populations, we used genome‐wide, single nucleotide polymorphisms (SNPs), produced using Diversity Array Technology sequencing (DArTseq) in the current study. This approach has been successfully used with minimal DNA sample requirements (Jaccoud et al., 2001) in numerous plant studies over the past decade (Pailles et al., 2017; Tang et al., 2015; Yang et al., 2016). We also investigated the genetic relatedness of *N. lloydii* to *N. microcarpa*. Furthermore, to understand the population genetic consequences of the rarity of *N. lloydii*, we undertook a comparative genetic analysis with one of other common species in the genus, *N. punctata* (Appendix 1: Figure A1), which occurs in sympatry with *N. lloydii* at some locations. Finally, because the reproductive phase (pollination, seed production, dispersal and survival) of a plant's life history can influence genetic diversity (Brown et al., 2003), we also conducted a two‐year study of life history reproductive traits of *N. lloydii*.

## 2 MATERIALS AND METHODS

### 2.1 Sample collection and genotyping

A total of 123 individuals of *Notelaea* from five species were sampled in this study (Table 1 and Appendix 1: Table A1). *Notelaea lloydii* was sampled from all known populations throughout the range of the species and the number of accessions from each population varied (from 1 to 17) depending on the population size and site accessibility. Samples of *N. microcarpa* were also obtained throughout its range to assess genetic similarity between *N. lloydii* and *N. microcarpa. Notelaea punctata* samples were collected from locations that are sympatric or nearby to *N. lloydii* populations for comparative analysis of genetic diversity. Fresh leaf material was collected from plants located in SE‐QLD where site access was granted and permits obtained (WIF418593017 and WITK18593017). Leaves were dried immediately in silica gel, and where fresh samples could not be obtained, dried leaf material from herbarium specimens was used.

**TABLE 1:** Details of the samples used during the study. All locations are in Queensland except for *N. microcarpa* sampled from New South Wales (NSW).

Species	Sample location	Population abbreviation	Number of samples
*N. lloydii*	Esk	LL_Esk	17
	Mt Crosby	LL_MtCr	17
	Lloyd Bird Park	LL_LB	16
	Moggill	LL_Moggill	16
	Kholo	LL_Kholo	5
	Mt Edwards	LL_MtEd	4
	Ebbw Vale	LL_Ebbw	1
*N. microcarpa*	Locker Valley	MCR_LV	8
	Central highlands	MCR_Other	2
	Chinchilla	MCR_Other	2
	Ipswich	MCR_Other	2
	Tablelands	MCR_Other	2
	Balonne	MCR_Other	1
	Charters Towers	MCR_Other	1
	Etheridge	MCR_Other	1
	Flinders	MCR_Other	1
	Goondiwindi	MCR_Other	1
	Murweh	MCR_Other	1
	North Burnett	MCR_Other	1
	Somerset	MCR_Other	1
	Toowoomba	MCR_Other	1
	New South Wales	MCR_NSW	2
*N. punctata*	Lloyd Bird Park	PNT_LB	10
	Samford	PNT_SERF	10

Genomic DNA was extracted as per methods described in Manawaduge et al. (2022), and DNA samples were sent to Diversity Arrays Technology Pty Ltd, Canberra, for DarTseq analysis. SNP callings were identified in each short sequence fragment (~20 bp) through proprietary DarT analytical pipelines as described in Tomkowiak et al. (2022), and the results of the SNP calls were returned as a matrix. DarTseq results were viewed and analysed using the DarTR package (Gruber et al., 2018) in the R platform (R Core Team, 2013). A single short sequence fragment with one SNP was considered as a locus, and the loci with call rates below 90% and average reproducibility below 95% were removed so that only high‐quality informative data were retained.

### 2.2 Spatial genetic structure and gene flow among *N. lloydii* populations

A principal coordinate analysis (PoA) was performed on SNP data using DarTR to visualise the genetic relationship between *N. lloydii* and *N. microcarpa*. A phylogenetic tree was also constructed using the maximum likelihood method as described in Manawaduge et al. (2022). Pairwise Wright's *F*_st_ between *N. lloydii* populations were estimated using StAMPP (Pembleton et al., 2013) in R. Nei's pairwise *F*_st_ (Nei, 1987) and D genetic distance (Nei, 1972) were also estimated using the hierfstat (Goudet, 2005) and StAMPP (Pembleton et al., 2013) packages in R, respectively. A Mantel test (Mantel, 1967) was performed using ade4 (Dray & Dufour, 2007) implemented in R to test the correlation between pairwise *F*_st_ and geographic distance. The LEA (Frichot & François, 2015) R package was used to estimate individual admixture coefficients by implementing a sparse, non‐negative matrix factorisation algorithm (sNMF) to estimate ancestry coefficients from large genotypic matrices and to evaluate the number of ancestral populations (*K*). The entire data set was run for *K* = 1 to 10, with 100 repetitions for each *K* value. The value of *K* that corresponded with the lowest cross‐entropy criterion was selected as the value that best explained the results (Frichot & François, 2015).

**FIGURE 1:**
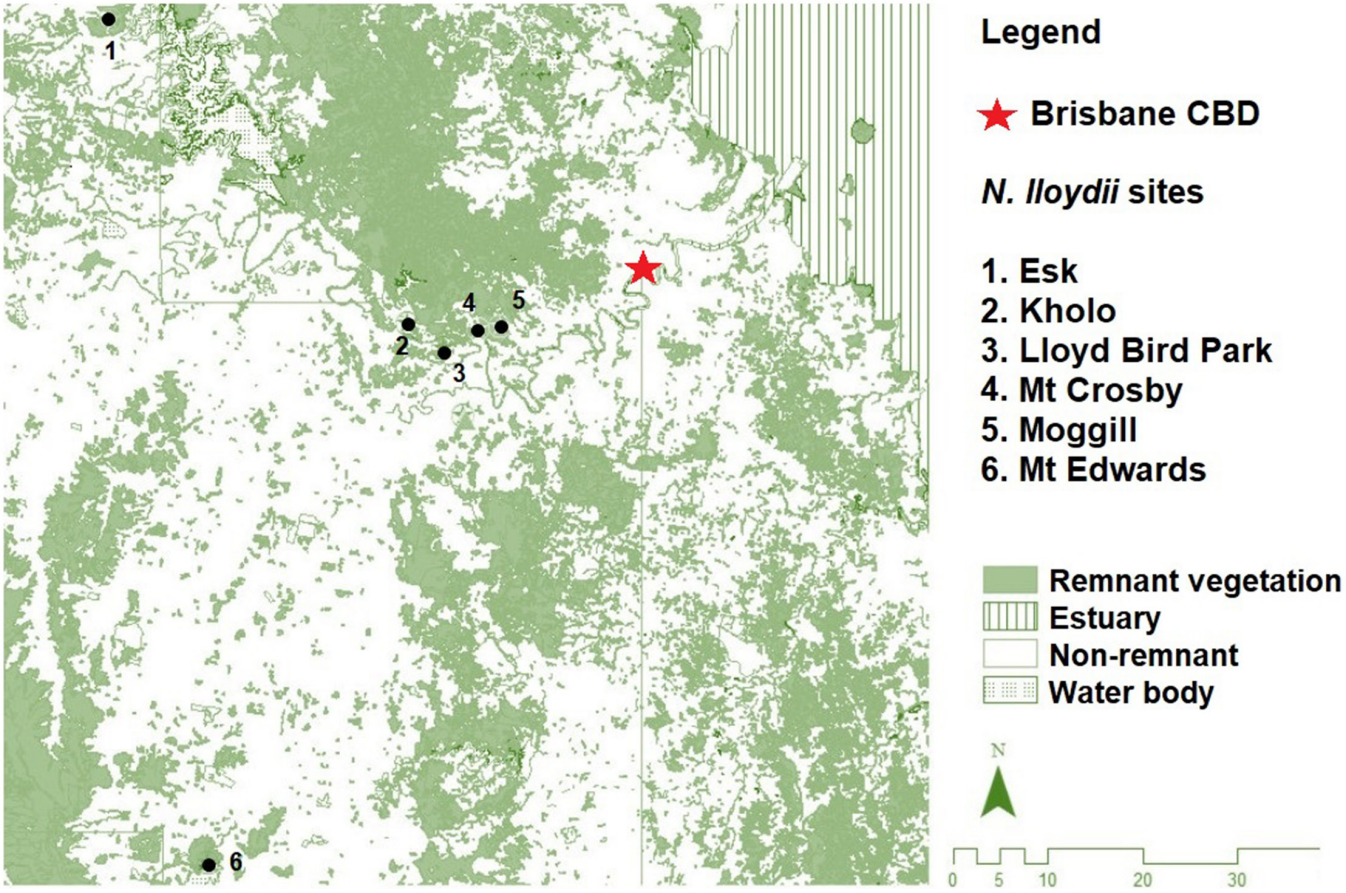
Map illustrating the extent of remnant vegetation cover (green shading) and the sampling locations of *N. lloydii* in south‐east Queensland, Australia.

CIRCUITSCAPE v 4.0.5 (McRae, 2006; McRae et al., 2008) was used to examine whether gene flow was a function of isolation by distance (IBD) or landscape resistance (IBR) associated with the cover of native vegetation (Figure 1) because native olive drupes are primarily dispersed by frugivorous vertebrates associated with forest vegetation. A remnant vegetation resistance surface (RS) was developed by transforming the Remnant Vegetation Cover (2017) vector layer (Queensland Spatial Catalogue: http://qldspatial.information.qld.gov.au/catalogue/) into a binary raster denoting the presence/absence of remnant vegetation at a cell size of 100 m. The RS was constrained to a 1 km buffer beyond the furthest sampling site in each direction. RS optimisation was carried out in R using ResistanceGA v4.0‐14 (Peterman, 2018). ResistanceGA uses a genetic algorithm (Scrucca, 2013) and linear mixed effects model with maximum likelihood population effects (MLPE; Clarke et al., 2002) implemented in lme4 (Bates et al., 2014) in R. Linear mixed effects models fitted with MLPE account for non‐independence among pairwise data (Clarke et al., 2002) and has been shown to perform better than alternative modelling techniques in landscape genetics model selection (Shirk et al., 2018). Optimisation of the remnant vegetation surface was performed using pairwise *F*_st_ as the response variable, and pairwise resistance distances were calculated in CIRCUITSCAPE using an eight‐neighbour connection scheme as the predictor variable. Two additional models, a distance model where every RS cell is set to a resistance of 1 and an intercept‐only model without any landscape structure, were also incorporated. Optimisation was performed twice to confirm convergence and the model performance was ranked based on Akaike Information Criterion (AIC), AIC corrected for small sample sizes (AICc) and for delta AICc (ΔAICc).

### 2.3 Comparative analysis of genetic diversity and relatedness of *N. lloydii* and *N. punctata*

Genetic diversity estimates were calculated from the SNP data for populations with a minimum of four individuals (six *N. lloydii* and two *N. punctata* populations). In SE‐QLD, only scattered trees of *N. microcarpa* were located and genotyped, and therefore, these individuals were not included in the genetic diversity analysis. Observed heterozygosity (*H*_o_), expected heterozygosity (*H*_e_) and inbreeding coefficient (*F*_IS_) for each locus within each population were estimated using the hierfstat (Goudet, 2005) in R, and population comparisons between these parameters using ANOVA were conducted in IBM SPSS Statistics for Windows, version 26.0. Pairwise relatedness (kinship coefficients) between individuals within a population and among populations were estimated using the maximum likelihood estimation method in SNPRelate (Zheng et al., 2012) implemented in R.

### 2.4 Analysis of reproductive life history traits

Flowering and fruiting of *N. lloydii* was examined over two consecutive years (2017–2019) at two sites in SE‐QLD (Lloyd Bird Park: LB‐Park and Moggill Conservation Park: Moggill). The number of florets and/or fruits on five mature trees at each site was recorded once every fortnight between October and March. Since *Notelaea* produce numerous axillary inflorescences with many florets, counts were made for only 10 small terminal branches on each tree. At the beginning of each flowering season, five branches were labelled and bagged with see‐through organza bags (225 × 165 mm^2^) before floral buds opened, while five other branches were labelled and left un‐bagged. For each species, the mean number of flowers per branch, flower to fruit conversion rates and fruit survival rates over the sampling period were calculated. The percentage of developed fruit loss was assumed to be due to fruit removal by frugivores. Therefore, the difference in fallen fruits between bagged and un‐bagged represents the number of fruits removed by frugivores, and percentage of fruit removal by frugivores was calculated using the equation; *% fruit removal by frugivores = % fruit loss in un‐bagged branches – % fruit loss in bagged branches*. Where assumptions of normality were violated, Kruskal‐Wallis non‐parametric tests followed by Dunn's multiple comparison *post‐hoc* tests were conducted using IBM SPSS Statistics for Windows, version 26.

The local distribution of *N. lloydii* (clumped, random or uniform) was assessed using the nearest‐neighbour method (Krebs, 1989) to provide insight into germination pattern. Since no boundary strip for the study area was considered and the sample size was small (*n* < 100), the Donnelly (1978) modification of the Clark and Evans (1954) test was used to calculate the standard normal deviate (*z*) in order to check the significance of deviation from randomness (Krebs, 1989).

## 3 RESULTS

### 3.1 Spatial genetic structure and gene flow among *N. lloydii* populations

**FIGURE 2:**
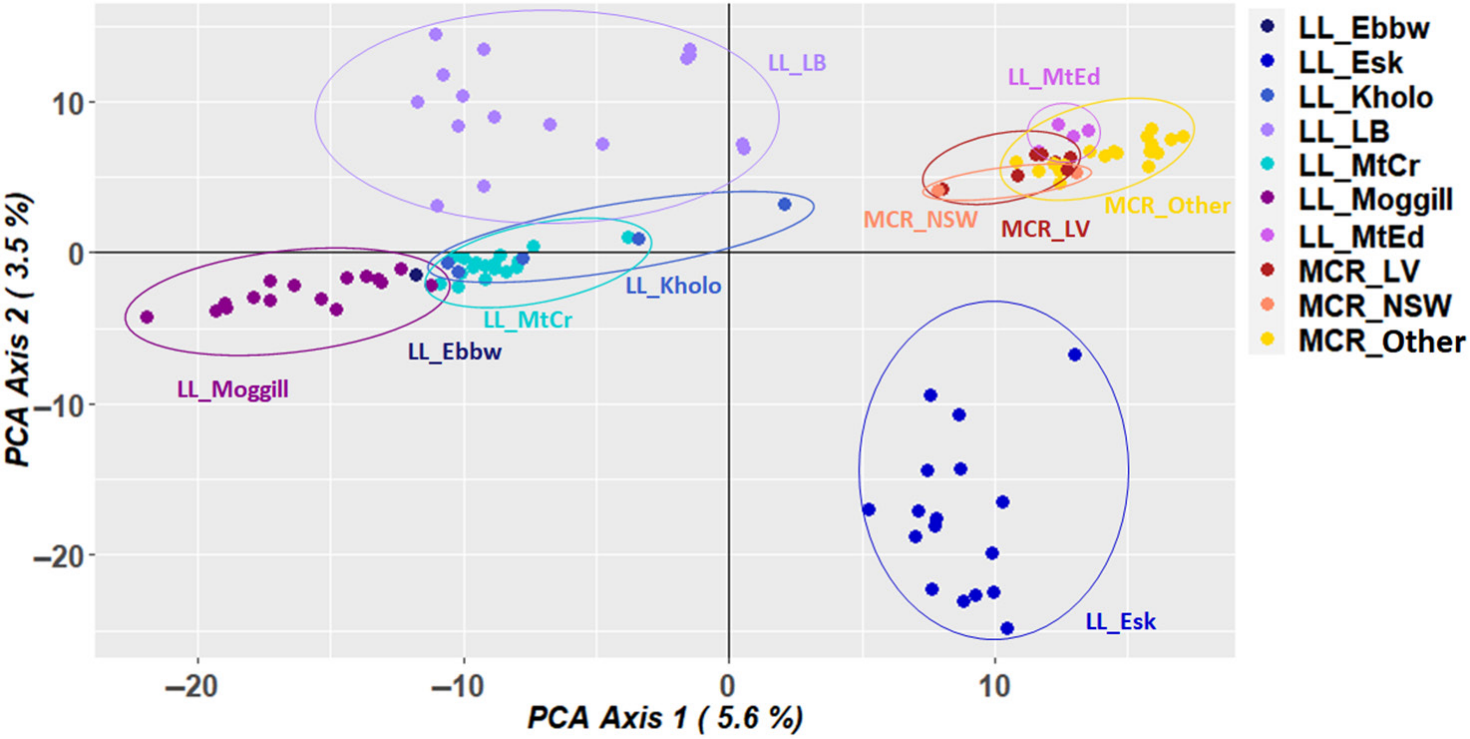
Scatter plot from the principal coordinate analysis of SNP data including all *N. lloydii* and *N. microcarpa* samples.

**FIGURE 3:**
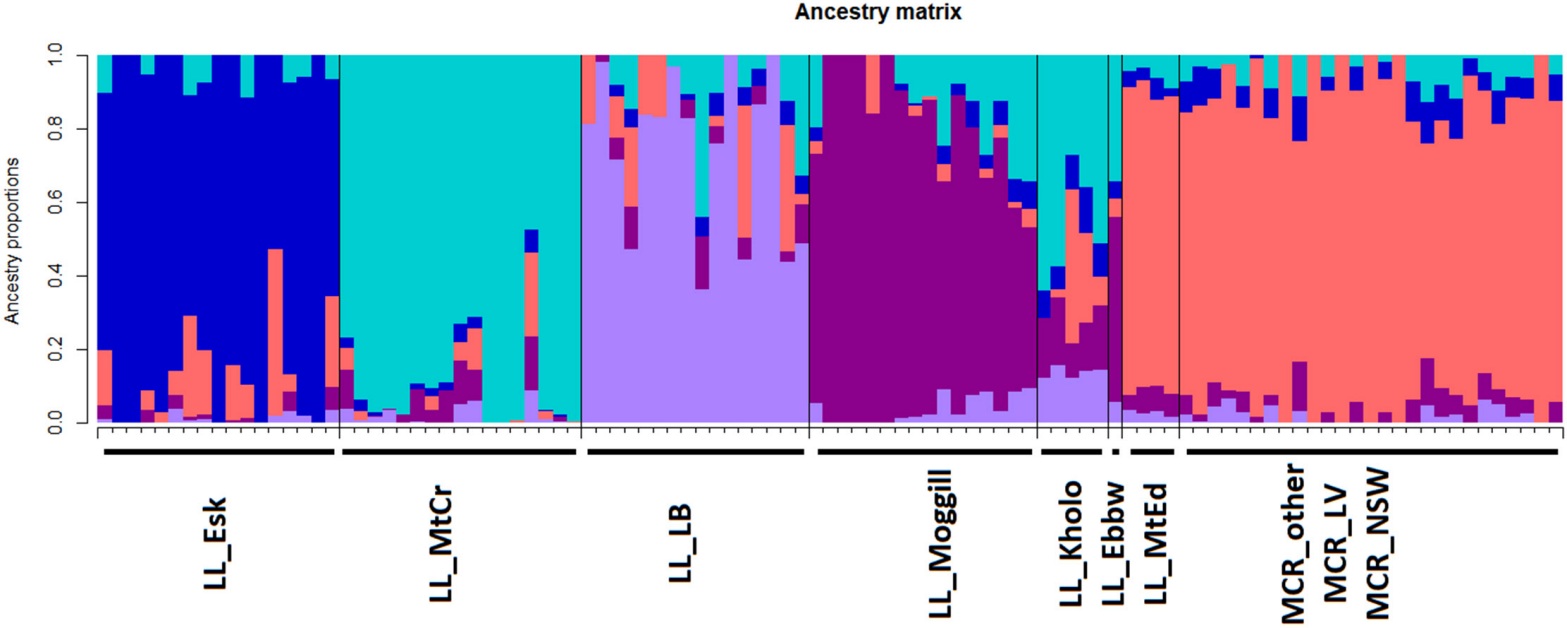
Estimates of admixture proportions of *N. lloydii and N. microcarpa*, inferred with sNMF using genome‐wide SNP data. Each bar is an individual and displays the relative assignment probability to each of five identified genetic groups.

**FIGURE A2:**
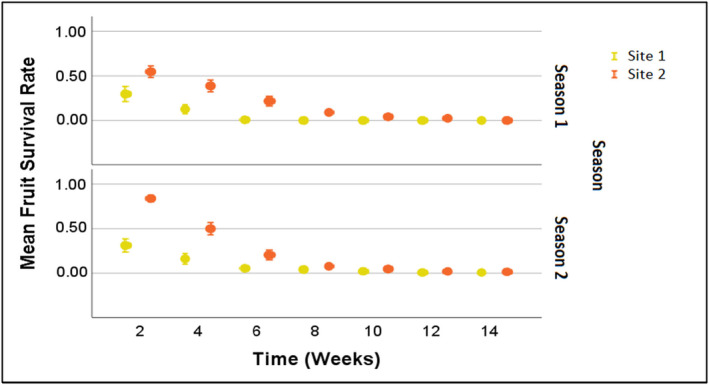
Error bar plots (error bars ±1 SE) of the average fruit survival rates of *N. lloydii* over time and between seasons for *N. lloydii*.

A PCoA was undertaken on the SNP data set containing 63,164 loci (call rate above 90%, average reproducibility above 95% and 3.7% missing data) for 103 individuals of *N. lloydii* and *N. microcarpa* (Figure 2). This included *N. lloydii* sampled from all known locations, *N. microcarpa* sampled from nearby locations in the Lockyer Valley region in Queensland (MCR_LV) and herbarium specimens to represent the wider distribution (MCR_other: from more distant locations in Queensland and MCR_NSW: from New South Wales). The first and second axes in the PCoA accounted for 5.6% and 3.6% of variation, respectively (Figure 2). All the *N. microcarpa* accessions cluster together indicating that there is little genetic variation among individuals, with overlap of samples from New South Wales and the Lockyer Valley in Queensland. *Notelaea lloydii* samples from Mt Edwards in Queensland also clearly overlap with *N. microcarpa*, indicating their close genetic affinity. Distinct clusters of *N. lloydii* samples from Esk and LB‐Park were also evident in the PCoA. Our phylogenetic tree also supports these groupings (Appendix 1: Figure A2). The admixture analysis of these data supported *K* = 5 (i.e., 5 groups) as the values corresponding with the lowest cross‐entropy from the sNMF algorithm in LEA (Figure 3). Almost all the samples of *N. microcarpa* across the eastern coast and a few *N. lloydii* cluster together, whereas the remaining *N. lloydii* samples exhibit some level of structure corresponding to sample location.

Pairwise Wright's *F*_st_ values and geographical distances are given in Table 2. *F*_st_ values revealed significant genetic differentiation between all *N. lloydii* populations. Mount Edwards and Esk populations of *N. lloydii* were most differentiated from all other populations (Table 2). A highly significant pattern of isolation‐by‐distance among *N. lloydii* populations was found (*R* = .8329, *p* = .0051). A comparison of *F*_st_ between sympatric populations of *N. lloydii* and *N. punctata* (at LB‐Park) revealed higher *F*_st_ values for *N. lloydii* (0.1379) than *N. punctata* (0.0592, Appendix 1: Table A2). Among the three landscape models tested, pairwise resistance distance and *F*_st_ showed strongest support based on ΔAICc for the distance model (Table 3). The remnant vegetation RS showed the least support, behind the intercept‐only model of no landscape structure.

**TABLE 2:** Pairwise Wright's *F*_st_ values (lower diagonal) and geographical (km) distances (upper diagonal) between the *N. lloydii* populations. Significantly different pairwise *F*_st_ comparisons (*p* < .05) are shown in bold.

	LL_Esk	LL_MtCr	LL_LB	LL_Moggill	LL_Kholo	LL_MtEd
LL_Esk	–	50.82	49.36	52.38	44.66	88.49
LL_MtCr	**0.0922**	–	4.19	2.46	7.47	62.5
LL_LB	**0.1059**	**0.0720**	–	6.57	4.51	59.2
LL_Moggill	**0.1212**	**0.0686**	**0.0934**	–	9.78	64.25
LL_Kholo	**0.0874**	**0.0439**	**0.0663**	**0.0763**	–	60.35
LL_MtEd	**0.1277**	**0.1300**	**0.1379**	**0.1686**	**0.1295**	–

**TABLE 3:** Results for landscape genetics resistance surface optimisation performed in ResistanceGA with surfaces ranked in order of support according to ΔAICc (Note: the “Resistance” column shows the assigned resistance values to the binary remnant vegetation layer where A denotes absence of the specified feature and P the presence).

Surface	AIC	AICc	ΔAICc	Resistance	Parameters (k)
Distance	−72.812	−69.466	0.000		2
Null	−64.711	−63.711	−5.756		1
Remnant vegetation	−73.466	−61.466	−8.000	A‐1; P‐2.5	3

### 3.2 Genetic diversity and relatedness within and between *N. lloydii* and *N. punctata* populations

Genetic diversity estimates were calculated for *N. lloydii* and *N. punctata* populations that were represented by at least four individuals. The final data set of *N. lloydii* consisted of 75 individuals from six populations and 54,757 loci, while *N. punctata* consisted of 20 individuals and 56,252 loci. Each data set contained 2.2% and 1.8% missing data (following removal of low quality and monomorphic loci), respectively. Mean genetic diversity measures (*H*_o_, *H*_e_ and *F*_is_) for each *N. lloydii* population clearly indicate that all populations exhibit significant (*p* < .001) inbreeding (Table 4). In every population, a significant (*p* < .001) homozygote excess was found (Appendix 1: Table A3) and *H*_o_ ranged from 0.0983 to 0.1242. *F*_is_ values were positive and ranged from 0.1261 to 0.1857. Average genetic diversity measures (*H*_o_, *H*_e_ and *F*_is_) for each *N. punctata* population revealed no evidence of inbreeding (Table 4). In every population, a significant (*p* < .001) heterozygote excess was found (Appendix 1: Table A3b) and *H*_o_ ranged from 0.2508 to 0.22525. *F*_is_ values were negative and ranged from −0.0557 to −0.0449.

**TABLE 4:** Mean observed heterozygosity (*H*_o_), expected heterozygosity (*H*_e_) and inbreeding coefficient (*F*_IS_) for each *N. lloydii* and *N. punctata* population (standard error of the mean is given in brackets).

Species	Population	*H*_o_	*H*_e_	*F*_is_
*N. lloydii*	LL_Esk	0.1141 (0.0006)	0.1471 (0.0007)	0.1857 (0.0019)
	LL_Kholo	0.1142 (0.0008)	0.1462 (0.0009)	0.1517 (0.0029)
	LL_LB	0.1242 (0.0007)	0.1467 (0.0007)	0.1261 (0.0018)
	LL_Moggill	0.1160 (0.0007)	0.1394 (0.0007)	0.1350 (0.0019)
	LL_MtCr	0.1197 (0.0007)	0.1494 (0.0007)	0.1590 (0.0017)
	LL_MtEd	0.0983 (0.0008)	0.1325 (0.0009)	0.1708 (0.0036)
	All	0.1144 (0.0003)	0.1537 (0.0009)	0.1436 (0.0003)
*N. punctata*	PNT_LB	0.2508 (0.0010)	0.2267 (0.0008)	−0.0557 (0.0017)
	PNT_SERF	0.2525 (0.0010)	0.2305 (0.0007)	−0.0449 (0.0017)
	All	0.2516 (0.0007)	0.2286 (0.0005)	−0.0502 (0.0012)

Pairwise relatedness between individuals revealed that average kinship coefficients (*k*) within each *N. lloydii* population were higher than within *N. punctata* populations (Table 5). The pairwise k of individuals within populations was higher than between populations. The highest average pairwise k within a location for *N. lloydii* was found at Mt Edwards (Table 5), where the two most related *N. lloydii* individuals were also identified with a kinship coefficient of 0.254 (Appendix 1: Table A4a–f).

**TABLE 5:** Average pairwise kinship values within (along the diagonal in bold) and between (lower diagonal) the populations of *N. lloydii* and *N. punctata.*

Species	Population	*N. lloydii*						*N. punctata*	
		Esk	MtCr	LB	Moggill	Kholo	MtEd	LB	SERF
*N. lloydii*	Esk	**0.0547**						–	–
	MtCr	0.0002	**0.0306**					–	–
	LB	0.0002	0.0013	**0.0472**				–	–
	Moggill	0.0000	0.0033	0.0010	**0.0566**			–	–
	Kholo	0.0013	0.0072	0.0042	0.0045	**0.0366**		–	–
	MtEd	0.0075	0.0015	0.0041	0.0005	0.0067	**0.1149**	–	–
*N. punctata*	LB	–	–	–	–	–	–	**0.0231**	
	SERF	–	–	–	–	–	–	0.0001	**0.0214**

### 3.3 Reproductive life history trait analysis

**FIGURE 4:**
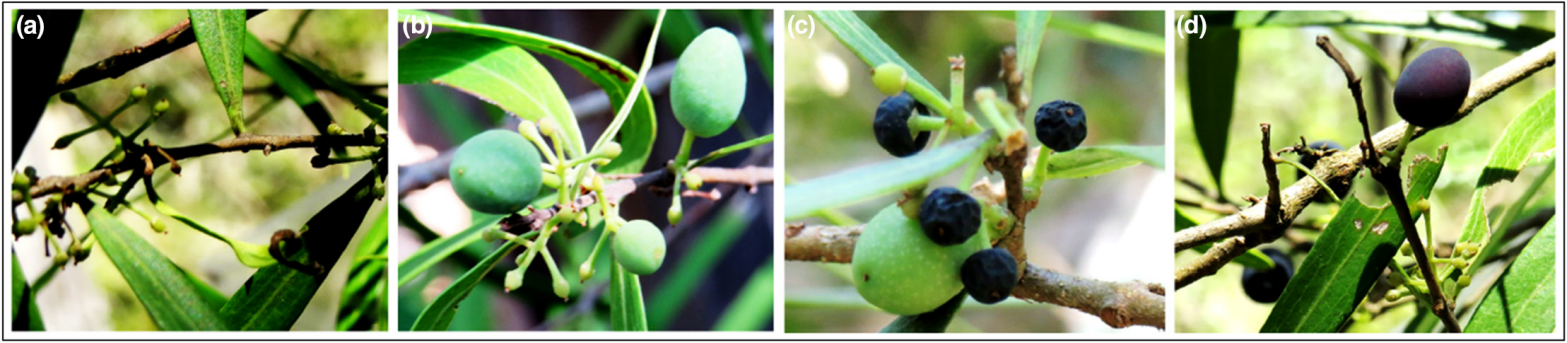
Fruit development stages in *N. lloydii* (a) fruit set/enlarged ovaries, (b) few mature fruits while others remain small, (c) some small fruits turn purple before maturity and (d) mature ripened fruits.

**FIGURE A3:**
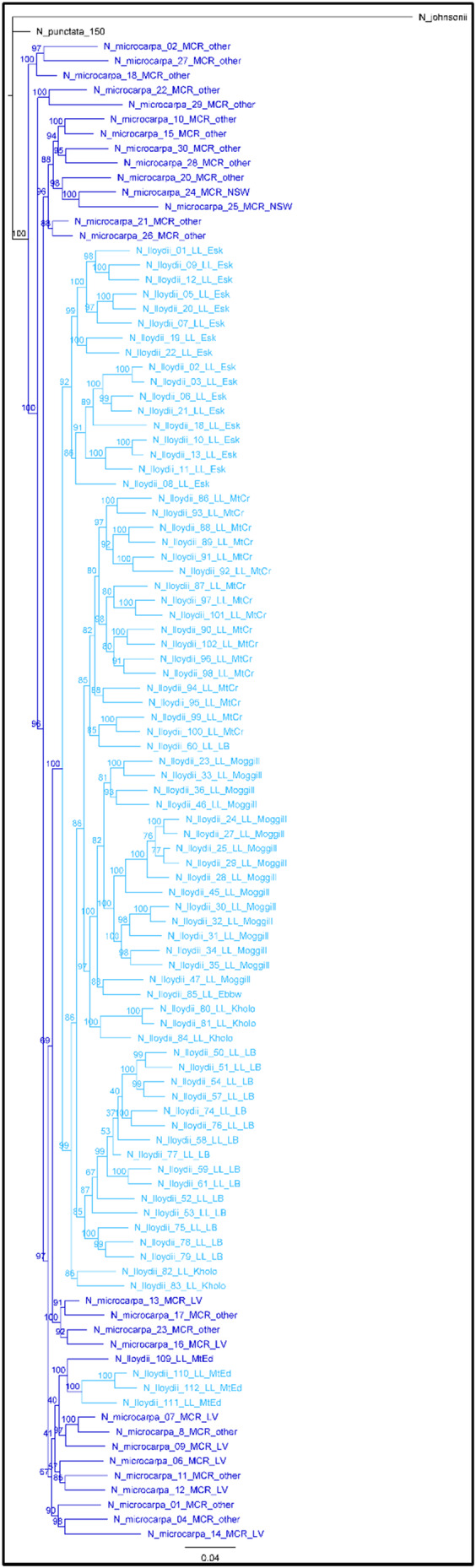
Maximum likelihood consensus tree, obtained from genomic SNPs, showing the phylogenetic relationships of *N. lloydii* (light blue) and *N. microcarpa* (dark blue). The numbers at each node represent the ultrafast‐bootstrap values.

A significant difference in the number of *N. lloydii* flowers produced in the two seasons (season 1—October 2017 to March 2018; season 2—October 2018 to March 2019) was found (Table 6). However, the flower to fruit conversion ratio for un‐bagged branches was not significantly different between the two seasons (*p* > .05). Not all immature fruits developed into mature fruits bearing seeds. Most of the small fruits at the initial stages remained small or excised (Figure 4). The mean survival rates over time were also not significantly different between the seasons, and the same pattern was observed between the two sites (Appendix 1: Figure A3). Nevertheless, the percentage of developed fruits was significantly higher in season 2, compared to season 1, in both bagged and un‐bagged branches (Table 6). While the percentage of fruit removal was lower in season 2 (3.7%) than in season 1 (26.0%), the number of fruits removed in season 2 was higher because the total number of developed fruits was higher than in season 1. Mean rainfall and temperature did not significantly differ between the two seasons (Appendix 1: Table A5). However, a comparison during the first month of the two seasons revealed that mean rainfall and mean maximum temperature were significantly different (Appendix 1: Table A5).

**TABLE 6:** Comparison of the average number of florets per branch, the average flower to fruit conversion rates and the percentage of the developed fruits from the initial fruits over the two seasons.

	Branch type	Group	Average	SD	*p*‐Value
The average number of florets per branch	Bagged	Season 1	64.20	50.99	.0330
		Season 2	46.38	28.22	
	Un‐bagged	Season 1	56.70	45.13	.0043
		Season 2	36.46	18.91	
The average flower to fruit conversion rates	Bagged	Season 1	0.77	0.29	.0001
		Season 2	0.51	0.34	
	Un‐bagged	Season 1	0.78	1.38	.0960
		Season 2	0.44	0.35	
The percentage of the developed fruits from the initial fruits	Bagged	Season 1	1.32	2.64	.0017
		Season 2	8.02	14.09	
	Un‐bagged	Season 1	0.96	2.59	.0319
		Season 2	4.79	11.35	

During season 1, less than 1% of the initial fruits developed into mature fruits on un‐bagged branches (Table 7). Even though slight variations were observed between bagged and unbagged branches, the difference was not significant (Appendix 1: Table A6). Thus, to check the exact fruit removal rates, only developed fruits were considered as they were the ones producing a fleshy mesocarp to attract seed dispersal agents and were viable. As shown in Table 7, the percentage loss of developed fruits was equal or higher for un‐bagged compared with bagged branches and this difference was due to fruit removal by frugivores.

**TABLE 7:** Comparison of the percentage fruit removal by frugivores over the two seasons.

Season	Site	% fruit loss		% removal by frugivores
		Bagged	Un‐bagged	
Season 1	Site 1	100.0	100.0	0.0
	Site 2	39.6	91.7	52.1
Season 2	Site 1	92.6	100.0	7.7
	Site 2	100.0	100.0	0.0

The nearest‐neighbour analysis in Moggill and LB‐Park revealed that at both sites, *N. lloydii* exhibited a clumped spatial distribution: the index of aggregation (*R*) was less than 1 (*R* = .437 and .592 for Moggill and LB‐Park, respectively) and the absolute value for *z* was greater than 1.96 (|*z*| = 4.673 and 5.100 for Moggill and LB‐Park, respectively).

## 4 DISCUSSION

### 4.1 Spatial genetic structure and gene flow

Small, fragmented plant populations may exhibit genetic differentiation facilitated by geographical and ecological factors, plant life history and reproductive traits and human impacts (Bezemer et al., 2019; Duminil et al., 2009; Huang et al., 2019; Loveless & Hamrick, 1984). In this study, all known populations of *N. lloydii* were sampled and distinct geographic clustering was found. In contrast, all the *N. microcarpa* samples clustered together with overlap of samples from New South Wales and the Lockyer Valley in Queensland, indicating little genetic differentiation. A significant finding was that *N. lloydii* samples from Mt Edwards in Queensland clearly overlapped with *N. microcarpa*, indicating close genetic affinity and suggesting that further investigation is required to determine whether they constitute two separate species.

Our analysis of genetic structure indicated moderate (*F*_st_ = 0.05–0.15) to high (*F*_st_ = 0.15–0.25) genetic differentiation and restricted gene flow between *N. lloydii* populations separated by less than seven kilometres. Furthermore, the population genetic structure of *N. lloydii* exhibited a pattern of isolation by distance rather than landscape resistance associated with remnant vegetation cover. Isolation by distance is often found in species with low dispersal ability combined with habitat specificity (Cipollini et al., 2017). While *N. lloydii* produces small drupes (Guymer, 1987) which may be easily dispersed long distances by frugivores, juvenile plants are rarely encountered, thus suggesting that recruitment by seedling germination is low and may be habitat‐specific. Furthermore, population structure can be influenced by plant life‐history traits such as the mating system, with self‐compatible species likely to experience higher population differentiation via inbreeding (Cipollini et al., 2017; Foxe et al., 2010; St Onge et al., 2011). Interestingly, our surveys of flowering indicate that *N. lloydii* is self‐compatible, but that fruit survival is low. In addition, founder effects at the time of colonisation can also lead to pronounced population structure (Loveless & Hamrick, 1984). Owing to their life history, annual plants typically experience strong founder effects, but high levels of gene flow ameliorate these effects. However, in woody perennials, recovery is slow due to their long‐life cycle (Austerlitz et al., 2000). In this instance, founder effects may result in reduced genetic diversity and strong population differentiation, and this may be the case for *N. lloydii*.

### 4.2 Genetic diversity and relatedness

The genetic diversity of a given population could vary depending on the plant's life‐history characteristics (Hamrick & Godt, 1996), and determining whether a specific level of genetic diversity is low or high can be challenging. Complicating matters further, genetic diversity measures have been calculated using various molecular markers (SSR, ALFP, SNP etc.), making the task more complex. Nevertheless, comparisons of genetic diversity estimated using similar molecular markers (e.g., SNPs), between closely related species, can provide valuable insight. In this study, the genetic diversity (i.e., expected heterozygosity) of the populations of both *Notelaea* species ranged from 0.13 to 0.23, which was relatively lower than that observed for other Oleaceae species with similar life history traits. Belaj et al. (2012) used SNPs to examine the diversity conserved in the World Olive Germplasm Bank (WOGB) located in Spain and found high genetic diversity (*H*_e_ = 0.442) for *Olea europaea* L. (Oleaceae), while Lee et al. (2022) conducted a population genomic study of an endangered shrub, *Abeliophyllum distichum* Nakai (Oleaceae), endemic to South Korea, and reported a mean expected heterozygosity of 0.319, using SNPs makers. In our study, we restricted our sampling of *N. punctata* populations to where they occurred in sympatry with *N. lloydii* in SE‐QLD because it is recommended in studies of rarity that both rare and common species should be sampled from the same geographical area (Gibson et al., 2008). However, as a result it is possible that the relatively low genetic diversity we observed for *N. punctata* could be the result of inadequate sampling, as this species has a widespread distribution.

Many studies have shown that rare and geographically restricted species tend to have lower genetic diversity than common, widespread congeners (Cole, 2003; Gibson et al., 2008; Gitzendanner & Soltis, 2000; Hamrick & Godt, 1996). Similarly, we found significantly lower genetic diversity within and among *N. lloydii* populations compared with the common and widespread (throughout SE‐QLD) *N. punctata*. All populations of *N. lloydii* exhibited lower levels of observed heterozygosity than expected heterozygosity indicating higher inbreeding. The opposite pattern was found for *N. punctata* populations; they exhibited significant heterozygosity excess and no evidence of inbreeding. Inbreeding in *N. lloydii* populations may be the result of small population size and restricted gene flow due to isolation by distance. However, the comparative analysis of genetic diversity between sympatric *N. lloydii* and *N. punctata* populations suggests that the reproductive biology of the two species may be different and that mechanisms linked to successful founder events may be important determinants of genetic diversity and inbreeding.

Pairwise kinship coefficients between individuals can be used to determine the degree of relatedness within and between populations and to detect founder effects during colonisation. Biparental inbreeding is known to be an unavoidable consequence in small, isolated populations in which individuals are clustered together (Sweigart et al., 1999). However, in each *N. lloydii* population, only a few individuals showed a half‐sib relationship, and the mean pairwise kinship coefficient was low, indicating that most individuals were unrelated. In general, pairwise kinship coefficients of individuals within a population were greater than between individuals of different populations, indicating some degree of founder effect, which could explain the observed structure and low genetic diversity.

Analysis of the distribution of individual *N. lloydii* plants at two sites revealed a clumping pattern of plants. In these sites, the individuals appeared to be of similar age, and only a few juvenile plants were found. Also, no germination was observed during preliminary seed germination tests (data not shown), and hence, seed germination and seedling establishment might be limited for *N. lloydii*. Therefore, the clumped distribution could be due to vegetative regeneration/propagation from lignotubers. However, if this was the cause of clumping, individual plants would be expected to have an identical genetic constitution, which was not the case, as very low genetic relatedness was generally found. This indicates that clumps of *N. lloydii* most likely arise by germination from a frugivore depositing seeds in a localised area. While some lignotubers were noted in this study (personal observation), these resulted mainly from the destruction of the above‐ground parts, usually by fire.

Low genetic diversity and inbreeding in small populations can lead to inbreeding depression (Ellstrand & Elam, 1993; Keller & Waller, 2002) which affects many different components of fitness in plants, such as plant size and growth, resistance to stress, seed yield, germination rate etc. (Charlesworth & Charlesworth, 2017; Frankham et al., 2002; Husband & Schemske, 1996; Keller & Waller, 2002; Naito et al., 2005). Even though theoretical models and empirical observations suggest that the magnitude of inbreeding depression is lower in habitually selfing plants because deleterious recessive alleles are expressed and purged through selection, some highly inbred species can maintain considerable levels of inbreeding depression (Husband & Schemske, 1996; Naito et al., 2005). Given the high level of inbreeding detected among proximate populations of *N. lloydii*, it is critical to evaluate whether inbreeding depression is impacting on the reproductive life history of the species, so that appropriate conservation measures can be developed.

### 4.3 Reproductive life history trait analysis

*Notelaea lloydii* produced flowers and fruits in two consecutive seasons but despite having many flowers, only a few *N. lloydii* fruits survived to maturity. Two to three days after full opening, some florets were naturally excised from the pedicel, while for most florets the petals wilted and fell off, but the enlarged ovaries remained attached to the pedicels. While these enlarged ovaries indicate early‐stage fruit development, surprisingly only about 1% developed into large, dark purple ripened fruits. Most of these enlarged ovaries/immature fruits were either excised 3–4 weeks later or remained as is for the duration of the season, raising doubts as to whether they were successfully pollinated and fertilised or had undergone selective fruit/embryo abortion.

Plant reproductive success is a function of both intrinsic and extrinsic biotic and abiotic factors (Abdala‐Roberts et al., 2014), and in our study, local weather conditions clearly impacted the reproductive output of *N. lloydii*. The percentage of developed fruits was significantly higher in the second season (about 5% on un‐bagged branches during Sep 2018—Mar 2019) relative to the first season (about 1% on un‐bagged branches during Sep 2017—Mar 2018). Mean rainfall and temperature during the first month of the flowering season (September) was significantly different between seasons—it was dry and hot in 2017 but comparatively wet and cool in 2018. However, regardless of the improved weather conditions in the second season, the percentage of developed fruits was only 5%, indicating high fruit abortion. Early high fruit abortion rates can be due to a range of reasons including lack of pollinators, insect attack or inbreeding depression (Simiqueli et al., 2018). However, in our study the average number of florets per branch, flower to fruit conversion rates and the percentage of developed fruits were not significantly different between bagged and un‐bagged branches. Therefore, the impact of pollinator availability and insect attack on *N. lloydii* was negligible and the high fruit abortion rate may be due to inbreeding depression and/or suboptimal conditions.

Interestingly, regardless of the high number of initial florets, some *N. lloydii* plants did not produce any mature fruit, indicating that these individuals may undergo high selective fruit abortion. Furthermore, no germination was observed during preliminary seed germination tests (data not shown), and only a few juvenile plants were found at each study site (personal observation). It is possible that certain environmental and/or climatic conditions are required for mass flowering, fruiting and seed germination in this species. For some plants, flowering and seed production can be highly variable between years and/or synchronising within years among individuals within a population, which is known as masting (Koenig et al., 2015). Future field observations are required to identify such processes, if occurring, and any significant changes in the long‐term reproductive success of *N. lloydii* in SE‐QLD.

### 4.4 Conservation implications

The results of our recent molecular systematics study indicate that *N. lloydii* and *N. microcarpa* do not show reciprocal monophyly (Manawaduge et al., 2022), but they do exhibit distinguishing growth forms and leaf morphologies (Guymer, 1987). Because leaf size and shape are functionally significant, adaptive traits (Nicotra et al., 2011; Read et al., 2014), even if *N. microcarpa* and *N. lloydii* constitute a single species, populations with different leaf form may be worthy of conservation. However, conservation policies do not encourage the listing of infraspecific ranks such as forms, morphs, or subvarieties (IUCN, 2012) under threat categories. It may be more appropriate to apply the concept of management units (Moritz, 1994) for populations that do not show reciprocal monophyly yet exhibit low levels of gene flow and are functionally independent. Results of this study indicate that the populations of *N. lloydii* are small, inbred and genetically isolated, placing them at risk of localised extinction as a result of stochastic events (genetic, demographic, environmental). It is particularly concerning that two proximate populations (approximately 6.5 km apart) located in protected areas (Moggill and Lloyd Bird Park) and connected by remnant vegetation, exhibit significant, moderate genetic structure. While isolated plants exist outside of protected areas, ongoing habitat disturbance, loss and fragmentation is a major threat in this rapidly urbanising region. Our results indicate that SE‐QLD populations represent unique management units that require local conservation management.

## 5 CONCLUSIONS

This study has identified significant genetic structure and low genetic diversity in the populations of *N. lloydii*. We conclude that founder effects are the most likely explanation for the low genetic diversity and significant genetic structure observed in SE‐QLD and unlike other woody perennials (Austerlitz et al., 2000), *N. lloydii* seems unsuccessful in maintaining adequate gene flow to overcome such effects. At this stage, it is not clear if such failure is due to lack of pollination and/or seed dispersal. Furthermore, inbreeding depression in both early and late reproductive stages may be occurring. Given ongoing threats associated with urbanisation, we recommend conservation management of SE‐QLD populations to prevent localised extinction. We also recommend further investigation of the population genetics of *N. lloydii* and *N. microcarpa* across their complete geographical range, combined with ecological studies to identify any functional or life history differences, and to better understand long‐term reproductive output.

## AUTHOR CONTRIBUTIONS

**Chapa G. Manawaduge:** Conceptualization (equal); data curation (lead); formal analysis (lead); funding acquisition (equal); investigation (equal); methodology (equal); writing – original draft (lead). **James Ryan:** Formal analysis (equal); writing – original draft (supporting). **Matthew J. Phillips:** Conceptualization (equal); investigation (equal); methodology (equal); supervision (equal); writing – review and editing (equal). **Susan Fuller:** Conceptualization (equal); funding acquisition (equal); investigation (equal); supervision (lead); writing – review and editing (equal).

### OPEN RESEARCH BADGES

This article has earned Open Data and Open Materials badges. Data and materials are available at https://doi.org/10.5061/dryad.hmgqnk9pb.

## Data Availability

The data are deposited in DRYAD and can be accessed via the link https://doi.org/10.5061/dryad.hmgqnk9pb.

## APPENDIX 1

**TABLE A1:** Details of the samples used in population genetics analysis.

Species	Sample ID	Location/population
*N. lloydii*	N_lloydii_01	Esk
	N_lloydii_02	Esk
	N_lloydii_03	Esk
	N_lloydii_05	Esk
	N_lloydii_06	Esk
	N_lloydii_07	Esk
	N_lloydii_08	Esk
	N_lloydii_09	Esk
	N_lloydii_10	Esk
	N_lloydii_11	Esk
	N_lloydii_12	Esk
	N_lloydii_13	Esk
	N_lloydii_18	Esk
	N_lloydii_19	Esk
	N_lloydii_20	Esk
	N_lloydii_21	Esk
	N_lloydii_22	Esk
	N_lloydii_23	Moggill
	N_lloydii_24	Moggill
	N_lloydii_25	Moggill
	N_lloydii_27	Moggill
	N_lloydii_28	Moggill
	N_lloydii_29	Moggill
	N_lloydii_30	Moggill
	N_lloydii_31	Moggill
	N_lloydii_32	Moggill
	N_lloydii_33	Moggill
	N_lloydii_34	Moggill
	N_lloydii_35	Moggill
	N_lloydii_36	Moggill
	N_lloydii_45	Moggill
	N_lloydii_46	Moggill
	N_lloydii_47	Moggill
	N_lloydii_50	Lloyd Bird Park
	N_lloydii_51	Lloyd Bird Park
	N_lloydii_52	Lloyd Bird Park
	N_lloydii_53	Lloyd Bird Park
	N_lloydii_54	Lloyd Bird Park
	N_lloydii_57	Lloyd Bird Park
	N_lloydii_58	Lloyd Bird Park

	N_lloydii_59	Lloyd Bird Park
	N_lloydii_60	Lloyd Bird Park
	N_lloydii_61	Lloyd Bird Park
	N_lloydii_74	Lloyd Bird Park
	N_lloydii_75	Lloyd Bird Park
	N_lloydii_76	Lloyd Bird Park
	N_lloydii_77	Lloyd Bird Park
	N_lloydii_78	Lloyd Bird Park
	N_lloydii_79	Lloyd Bird Park
	N_lloydii_80	Kholo
	N_lloydii_81	Kholo
	N_lloydii_82	Kholo
	N_lloydii_83	Kholo
	N_lloydii_84	Kholo
	N_lloydii_85	Ebbw
	N_lloydii_86	Mt Crosby
	N_lloydii_87	Mt Crosby
	N_lloydii_88	Mt Crosby
	N_lloydii_89	Mt Crosby
	N_lloydii_90	Mt Crosby
	N_lloydii_91	Mt Crosby
	N_lloydii_92	Mt Crosby
	N_lloydii_93	Mt Crosby
	N_lloydii_94	Mt Crosby
	N_lloydii_95	Mt Crosby
	N_lloydii_96	Mt Crosby
	N_lloydii_97	Mt Crosby
	N_lloydii_98	Mt Crosby
	N_lloydii_99	Mt Crosby
	N_lloydii_100	Mt Crosby
	N_lloydii_101	Mt Crosby
	N_lloydii_102	Mt Crosby
	N_lloydii_109	Mt Edwards
	N_lloydii_110	Mt Edwards
	N_lloydii_111	Mt Edwards
N_lloydii_112	Mt Edwards
*N. punctata*	N_punctata_101	Lloyd Bird Park
	N_punctata_102	Lloyd Bird Park
	N_punctata_103	Lloyd Bird Park
	N_punctata_104	Lloyd Bird Park
	N_punctata_105	Lloyd Bird Park
	N_punctata_106	Lloyd Bird Park
	N_punctata_108	Lloyd Bird Park
	N_punctata_109	Lloyd Bird Park
	N_punctata_110	Lloyd Bird Park
	N_punctata_111	Lloyd Bird Park
	N_punctata_S1	Samford

	N_punctata_S2	Samford
	N_punctata_S4	Samford
	N_punctata_S5	Samford
	N_punctata_S6	Samford
	N_punctata_S7	Samford
	N_punctata_S8	Samford
	N_punctata_S10	Samford
	N_punctata_S11	Samford
N_punctata_S12	Samford
*N. microcarpa*	N_microcarpa_01	Somerset
	N_microcarpa_02	Tablelands
	N_microcarpa_03	Gladstone
	N_microcarpa_04	North Burnett
	N_microcarpa_06	Locker valley
	N_microcarpa_07	Locker valley
	N_microcarpa_08	Locker valley
	N_microcarpa_09	Locker valley
	N_microcarpa_10	Chinchilla
	N_microcarpa_11	Ipswich
	N_microcarpa_12	Locker valley
	N_microcarpa_13	Locker valley
	N_microcarpa_14	Locker valley
	N_microcarpa_15	Chinchilla
	N_microcarpa_16	Locker valley
	N_microcarpa_17	Ipswich
	N_microcarpa_18	Tablelands
	N_microcarpa_19	Tablelands
	N_microcarpa_20	Goondiwindi
	N_microcarpa_21	Central highlands
	N_microcarpa_22	Flinders
	N_microcarpa_23	Murweh
	N_microcarpa_24	Tenterfield (NSW)
	N_microcarpa_25	NSW
	N_microcarpa_26	Central highlands
	N_microcarpa_27	Etheridge
	N_microcarpa_28	Toowoomba
	N_microcarpa_29	Charters towers
	N_microcarpa_30	Balonne

**TABLE A2:** Pairwise *F*_st_ values (lower diagonal) and geographical distances (km, upper diagonal) among the *N. punctata* populations.

	LO_LB	LO_SERF
LO_LB		59.19
LO_SERF	0.0272	

**TABLE A3:** ANOVA results comparing *H*_o_, *H*_e_ and *F*_is_ among and within *N. lloydii* and *N. punctata* populations.

Species	Parameter	df	*F*	Sig.
*N. lloydii*	*H*_o_	328,507	149.290	.000
	*H*_e_	328,206	64.590	.000
	*F*_is_	168,341	118.431	.000
*N. punctata*	*H*_o_	112,502	1.422	.233
	*H*_e_	112,502	13.038	.000
	*F*_is_	89,430	20.005	.000
*N. lloydii* & *N. punctata*	*H*_o_	441,015	42954.536	.000
	*H*_e_	440,714	18017.997	.000
	*F*_is_	257,777	18887.046	.000

**TABLE A4:** (a) Pairwise kinship values between (lower diagonal) the individuals from Esk. (b) Pairwise kinship values between (lower diagonal) the individuals from Mount Crosby. (c) Pairwise kinship values between (lower diagonal) the individuals from Lloyd Bird Park. (d) Pairwise kinship values between (lower diagonal) the individuals from Moggill. (e) Pairwise kinship values between (lower diagonal) the individuals from Kholo. (f) Pairwise kinship values between (lower diagonal) the individuals from Mount Edwards.

(a). Esk	N_lloydii_01	N_lloydii_02	N_lloydii_03	N_lloydii_05	N_lloydii_06	N_lloydii_07	N_lloydii_08	N_lloydii_09	N_lloydii_10	N_lloydii_11	N_lloydii_12	N_lloydii_13	N_lloydii_18	N_lloydii_19	N_lloydii_20	N_lloydii_21
N_lloydii_02	0.044															
N_lloydii_03	0.081	0.222														
N_lloydii_05	0.056	0.032	0.066													
N_lloydii_06	0.040	0.127	0.093	0.037												
N_lloydii_07	0.049	0.055	0.058	0.099	0.059											
N_lloydii_08	0.027	0.070	0.046	0.033	0.050	0.035										
N_lloydii_09	0.076	0.028	0.038	0.108	0.057	0.041	0.030									
N_lloydii_10	0.037	0.059	0.054	0.049	0.073	0.081	0.028	0.026								
N_lloydii_11	0.028	0.062	0.052	0.018	0.084	0.032	0.026	0.026	0.100							
N_lloydii_12	0.088	0.027	0.064	0.112	0.033	0.049	0.023	0.132	0.055	0.022						
N_lloydii_13	0.042	0.094	0.059	0.023	0.091	0.066	0.021	0.025	0.233	0.171	0.026					
N_lloydii_18	0.010	0.059	0.033	0.005	0.046	0.012	0.020	0.013	0.021	0.033	0.013	0.025				
N_lloydii_19	0.029	0.044	0.059	0.056	0.030	0.069	0.028	0.029	0.085	0.036	0.054	0.075	0.010			
N_lloydii_20	0.049	0.030	0.043	0.163	0.040	0.072	0.031	0.059	0.061	0.032	0.085	0.077	0.009	0.076		
N_lloydii_21	0.033	0.159	0.105	0.034	0.144	0.080	0.033	0.021	0.090	0.088	0.024	0.126	0.063	0.075	0.057	
N_lloydii_22	0.033	0.014	0.044	0.036	0.022	0.023	0.020	0.022	0.041	0.041	0.058	0.030	0.010	0.068	0.049	0.020

(b). MtCr	N_lloydii_86	N_lloydii_87	N_lloydii_88	N_lloydii_89	N_lloydii_90	N_lloydii_91	N_lloydii_92	N_lloydii_93	N_lloydii_94	N_lloydii_95	N_lloydii_96	N_lloydii_97	N_lloydii_98	N_lloydii_99	N_lloydii_100	N_lloydii_101
N_lloydii_87	0.018															
N_lloydii_88	0.033	0.019														
N_lloydii_89	0.027	0.007	0.118													
N_lloydii_90	0.018	0.039	0.015	0.015												
N_lloydii_91	0.022	0.007	0.053	0.034	0.006											
N_lloydii_92	0.017	0.018	0.059	0.043	0.018	0.114										
N_lloydii_93	0.066	0.019	0.053	0.031	0.029	0.061	0.015									
N_lloydii_94	0.015	0.029	0.020	0.026	0.024	0.020	0.027	0.007								
N_lloydii_95	0.017	0.018	0.021	0.011	0.014	0.007	0.023	0.009	0.019							
N_lloydii_96	0.011	0.047	0.025	0.010	0.075	0.049	0.071	0.034	0.019	0.014						
N_lloydii_97	0.012	0.069	0.013	0.068	0.053	0.013	0.021	0.020	0.021	0.018	0.058					
N_lloydii_98	0.022	0.073	0.037	0.004	0.048	0.012	0.034	0.052	0.032	0.030	0.084	0.085				
N_lloydii_99	0.006	0.006	0.004	0.002	0.003	0.005	0.007	0.002	0.007	0.006	0.008	0.003	0.003			
N_lloydii_100	0.012	0.024	0.013	0.013	0.042	0.005	0.025	0.048	0.014	0.020	0.068	0.025	0.073	0.119		
N_lloydii_101	0.014	0.019	0.035	0.108	0.020	0.024	0.021	0.010	0.025	0.022	0.018	0.140	0.019	0.003	0.009	
N_lloydii_102	0.009	0.052	0.014	0.009	0.098	0.008	0.023	0.026	0.016	0.029	0.056	0.095	0.082	0.006	0.048	0.023

(c). Lloyd bird	N_lloydii_50	N_lloydii_51	N_lloydii_52	N_lloydii_53	N_lloydii_54	N_lloydii_57	N_lloydii_58	N_lloydii_59	N_lloydii_60	N_lloydii_61	N_lloydii_74	N_lloydii_75	N_lloydii_76	N_lloydii_77	N_lloydii_78
N_lloydii_51	0.120														
N_lloydii_52	0.025	0.069													
N_lloydii_53	0.024	0.035	0.018												
N_lloydii_54	0.113	0.118	0.044	0.019											
N_lloydii_57	0.113	0.115	0.048	0.016	0.126										
N_lloydii_58	0.062	0.108	0.043	0.024	0.076	0.106									
N_lloydii_59	0.053	0.075	0.030	0.055	0.046	0.042	0.094								
N_lloydii_60	0.005	0.017	0.013	0.016	0.004	0.000	0.036	0.023							
N_lloydii_61	0.032	0.064	0.046	0.023	0.034	0.025	0.071	0.134	0.038						
N_lloydii_74	0.070	0.134	0.087	0.041	0.091	0.079	0.110	0.086	0.016	0.081					
N_lloydii_75	0.012	0.016	0.036	0.015	0.010	0.014	0.011	0.017	0.007	0.025	0.037				
N_lloydii_76	0.067	0.113	0.047	0.025	0.056	0.063	0.091	0.068	0.014	0.059	0.111	0.010			
N_lloydii_77	0.064	0.088	0.037	0.023	0.058	0.056	0.101	0.065	0.020	0.069	0.109	0.012	0.093		
N_lloydii_78	0.019	0.010	0.018	0.013	0.016	0.011	0.009	0.020	0.003	0.028	0.032	0.073	0.023	0.025	
N_lloydii_79	0.006	0.027	0.028	0.008	0.007	0.010	0.034	0.046	0.011	0.029	0.036	0.079	0.021	0.027	0.082

(d). Moggill	N_lloydii_23	N_lloydii_24	N_lloydii_25	N_lloydii_27	N_lloydii_28	N_lloydii_29	N_lloydii_30	N_lloydii_31	N_lloydii_32	N_lloydii_33	N_lloydii_34	N_lloydii_35	N_lloydii_36	N_lloydii_45	N_lloydii_46
N_lloydii_24	0.043														
N_lloydii_25	0.039	0.220													
N_lloydii_27	0.034	0.217	0.230												
N_lloydii_28	0.013	0.132	0.211	0.124											
N_lloydii_29	0.036	0.133	0.229	0.156	0.106										
N_lloydii_30	0.041	0.045	0.086	0.050	0.031	0.074									
N_lloydii_31	0.088	0.052	0.075	0.060	0.023	0.058	0.075								
N_lloydii_32	0.053	0.028	0.078	0.044	0.034	0.066	0.198	0.082							
N_lloydii_33	0.096	0.017	0.021	0.021	0.011	0.024	0.039	0.040	0.038						
N_lloydii_34	0.027	0.061	0.123	0.060	0.051	0.073	0.078	0.075	0.086	0.041					
N_lloydii_35	0.048	0.035	0.048	0.028	0.020	0.034	0.069	0.069	0.046	0.053	0.095				
N_lloydii_36	0.048	0.030	0.042	0.021	0.022	0.034	0.035	0.029	0.039	0.063	0.067	0.053			
N_lloydii_45	0.032	0.052	0.100	0.061	0.050	0.068	0.056	0.054	0.048	0.041	0.051	0.044	0.031		
N_lloydii_46	0.049	0.020	0.026	0.021	0.018	0.019	0.044	0.038	0.031	0.040	0.026	0.050	0.039	0.026	
N_lloydii_47	0.031	0.014	0.019	0.017	0.009	0.013	0.023	0.044	0.022	0.042	0.025	0.023	0.040	0.028	0.030

(e). Kholo	N_lloydii_80	N_lloydii_81	N_lloydii_82	N_lloydii_83
N_lloydii_81	0.221			
N_lloydii_82	0.004	0.009		
N_lloydii_83	0.012	0.014	0.015	
N_lloydii_84	0.025	0.040	0.010	0.015

(f). MtEd	N_lloydii_109	N_lloydii_110	N_lloydii_111
N_lloydii_109			
N_lloydii_110	0.077		
N_lloydii_111	0.069	0.115	
N_lloydii_112	0.069	0.254	0.105

**TABLE A5:** Mean monthly rainfall, maximum temperature and minimum temperature over the two seasons.

	Season	Sep to Mar			September		
		Average	SD	*p*‐Value	Average	SD	*p*‐Value
Mean rainfall (mm)	Season 1	111.37	87.39	.0559	2.53	3.01	.0358
	Season 2	65.67	60.65		16.27	7.02	
Mean max temp (°C)	Season 1	29.40	1.87	.3492	28.43	1.21	.0480
	Season 2	30.08	2.66		25.80	1.08	
Mean min temp (°C)	Season 1	17.94	3.19	.8705	11.73	2.61	1.0000
	Season 2	18.11	3.57		11.73	2.20	

*Source*: The Bureau of Meteorology, Australia: http://www.bom.gov.au/.

**TABLE A6:** Flowering and fruiting differences between two treatments; bagged and unbagged.

	Group	Count	Sum	Average	SD	*F*	*p*‐Value
Mean number of florets per branch	B	50	3210	64.20	50.99	0.6066	.4379
	UB	50	2835	56.70	45.13		
Flower to fruits conversion rated	B	50	38.33	0.77	0.29	0.0066	.9357
	UB	50	39.14	0.78	1.38		
% developed fruits	B	48	63.49	1.32	2.64	0.4518	.5032
	UB	44	42.05	0.96	2.59		
